# Geographical accessibility and spatial coverage modeling of the primary health care network in the Western Province of Rwanda

**DOI:** 10.1186/1476-072X-11-40

**Published:** 2012-09-17

**Authors:** Ulises Huerta Munoz, Carina Källestål

**Affiliations:** 1 Department of Women’s and Children’s Health, International Maternal and Child Health (IMCH), Uppsala University, Uppsala, Sweden

## Abstract

**Background:**

Primary health care is essential in improving and maintaining the health of populations. It has the potential to accelerate achievement of the Millennium Development Goals and fulfill the “Health for All” doctrine of the Alma-Ata Declaration. Understanding the performance of the health system from a geographic perspective is important for improved health planning and evidence-based policy development. The aims of this study were to measure geographical accessibility, model spatial coverage of the existing primary health facility network, estimate the number of primary health facilities working under capacity and the population underserved in the Western Province of Rwanda.

**Methods:**

This study uses health facility, population and ancillary data for the Western Province of Rwanda. Three different travel scenarios utilized by the population to attend the nearest primary health facility were defined with a maximum travelling time of 60 minutes: Scenario 1 – walking; Scenario 2 – walking and cycling; and Scenario 3 – walking and public transportation. Considering these scenarios, a raster surface of travel time between primary health facilities and population was developed. To model spatial coverage and estimate the number of primary health facilities working under capacity, the catchment area of each facility was calculated by taking into account population coverage capacity, the population distribution, the terrain topography and the travelling modes through the different land categories.

**Results:**

Scenario 2 (walking and cycling) has the highest degree of geographical accessibility followed by Scenario 3 (walking and public transportation). The lowest level of accessibility can be observed in Scenario 1 (walking). The total population covered differs depending on the type of travel scenario. The existing primary health facility network covers only 26.6% of the population in Scenario 1. In Scenario 2, the use of a bicycle greatly increases the population being served to 58% of inhabitants. When considering Scenario 3, the total population served is 34.3%.

**Conclusions:**

Significant spatial variations in geographical accessibility and spatial coverage were observed across the three travel scenarios. The analysis demonstrates that regardless of which travel scenario is used, the majority of the population in the Western Province does not have access to the existing primary health facility network. Our findings also demonstrate the usefulness of GIS methods to leverage multiple datasets from different sources in a spatial framework to provide support to evidence-based planning and resource allocation decision-making in developing countries.

## Background

The 1978 Alma-Ata Declaration created a primary health care (PHC) revolution that embodied the principles of equity, social justice, and health for all. PHC “is the first level of contact of individuals, the family, and the community with the national health system bringing health care as close as possible to where people live and work, and constitutes the first element of a continuing health care process” [1]. More than 30 years later, the tenets of Alma-Ata remain relevant. PHC has both the potential to accelerate the achievement of the Millennium Development Goals (MDG) and fulfill the “Health for All” doctrine of the Alma-Ata Declaration by providing acceptable, accessible, appropriate, and affordable health care [2].

Many challenges remain, however, to achieving the goal of “Health for All” and the MDGs. Health systems consistently contribute to widening inequities in health. Access to health care is still governed by the inverse care law: the availability of good quality medical care tends to be inversely related to the need for it [3].

Access to health care services is multidimensional. In this paper, we use the conceptual framework described by Peters et al. [4]. The framework centers on the concept of quality of care and describes the following four dimensions (each of which has a supply and demand element): 1) Geographical accessibility – the physical distance or travel time between the service delivery point and the user; 2) Availability – the opportunity to access the right type of health care services when needed as well as having the appropriate type of service providers, materials, and equipment; 3) Financial accessibility – the relationship between the price of services and the willingness and ability of users to pay for those services, as well as protection from financial consequences of health expenses; and 4) Acceptability – the responsiveness of health service providers to the social and cultural expectations of individual users and communities. In this paper, we concentrate on the two dimensions that are spatial in nature: geographical accessibility and availability. In many parts of the developing world, factors that affect the availability of health services include: lack of infrastructure, medical equipment, and supplies; shortage of or inadequate drugs; lack of and unequal distribution of qualified health personnel; and weak capacity for planning, managing, and supervising human resources [5]. Geographical accessibility presents an important barrier to accessing health services. Studies in developing countries have demonstrated that physical proximity of health services is strongly linked to primary health care utilization [6-11].

In terms of health system performance, the spatial elements of availability and accessibilty can be converted to availability and accessibility coverage. Availability coverage demonstrates what resources are available and in what amount for delivering services. The availability of such resources limits the maximum capacity of the service and thus determines the amount of service that can be provided to the target population. Availability coverage relates the capacity of the health system to the size of the target population. Accessibility coverage determines how physically accessible resources are for the population [12]. Distance and time are both important factors of accessibility. The World Health Organization (WHO) recommends using travel time, rather than distance, to assess geographical accessibility. The vast differences in geography and transportation infrastructure amongst and within countries make measures of distance to health facilities difficult to compare [13]. In the case of accessibility coverage, the maximum capacity of the service is limited by the number of people who can reach and use it [12].

Combining availability and accessibility coverage allows us to define spatial coverage and to analyze, concurrently, the physical accessibility of the supply and the adequacy of the supply to cover the demand. Spatial coverage simultaneously takes into account the location and the maximum coverage capacity of each health facility, the geographical distribution of the population, the landscape through which the patient needs to cross to reach the health facility, and the mode of transportation [14].

Despite the adoption of pro-poor health policies and interventions by sub-Saharan African governments, health inequities and inaccessibility to basic health interventions remains high. It is imperative for resource-constrained countries in sub-Saharan Africa to monitor trends in health equity and access to essential PHC interventions to make the most efficient use of available resources and target those whose needs are greatest [15].

Advances in Geographical Information Systems (GIS) have contributed to more effective analyses of some aspects of health systems. GIS has been used to assess health care needs; analyze access to health services and understand disparities in access among different groups; evaluate health care utilization and its geographical variations; plan and evaluate health services; and provide spatial decision-making support for health care delivery [16].

This study utilizes GIS to measure geographical accessibility and spatial coverage of the public health system at the primary level in the Western Province (WP) of Rwanda. The objectives of this study were to measure geographical accessibility, model spatial coverage of the existing primary health facility network, estimate the number of primary health facilities working under capacity and the population underserved in the Western Province of Rwanda.

## Methods

### Study site

The decentralized, three-tiered public health system of Rwanda consists of central, intermediate and peripheral levels. The central level consists of the directorates of the Ministry of Health (MoH) and the national reference hospitals. The second level consists of 30 administrative districts, each of which contains a Health, Family Promotion, and Protection of Children’s Rights unit. Each administrative district has at least one district health hospital. The third tier consists of PHC facilities: health centres, health posts and dispensaries. Health centres provide a Minimum Package of Activities (MPA) which include: a) promotional services that include information, education, and communication, psychosocial support, nutritional activities, community participation, home visits, and hygiene and sanitation; b) preventive services that comprise premarital consultations, antenatal care (ANC), postpartum care for the mother and child, family planning counselling and services, school health, and epidemiologic surveillance activities; and c) curative services that cover consultations, management of chronically ill patients, nutritional rehabilitation, prescription or administration of medicines, observation before hospitalization, normal deliveries, minor surgical interventions, and laboratory testing. Health posts provide a reduced MPA which includes curative outpatient care, certain diagnostic tests, child immunization, growth monitoring for children under five years, ANC, family planning, and health education [17]. Health dispensaries provide primary health care and outpatient services, referral and outreach services that include immunization, family planning, growth monitoring and ANC [18].

The study area concerns the WP, which is situated next to Lake Kivu. A range of mountains stretches from north to south through the western area, making it the nation’s region of highest altitude. Altitudes range from 900 m in the southwest to 3000 in the highlands of the northwest and 4500 m in the regions of the Congo-Nile Crest and the chain of volcanoes [19]. The province was created in January of 2006 as part of the Government of Rwanda decentralization program that reorganized the country’s local government structures. The WP is divided into 7 districts (Cyangugu, Gasiza, Gisenyi, Kibuye, Ngororero, Nyamasheke, and Rutsiro) and 97 sub-districts called sectors. The capital of the WP is the city of Kibuye [20]. Based on the district baseline demographic and socioeconomic survey of 2008, the WP has a total population of 2,091,065 and a population density above 350/km^2^[21]. The population is mostly rural with 74% depending on subsistence agriculture for its livelihood [22]. The province is the second poorest region in the country and one of two provinces where food insecurity and increasing levels of inequality are concentrated [22,23].

### Datasets

#### Primary health facilities

**Figure 1:**
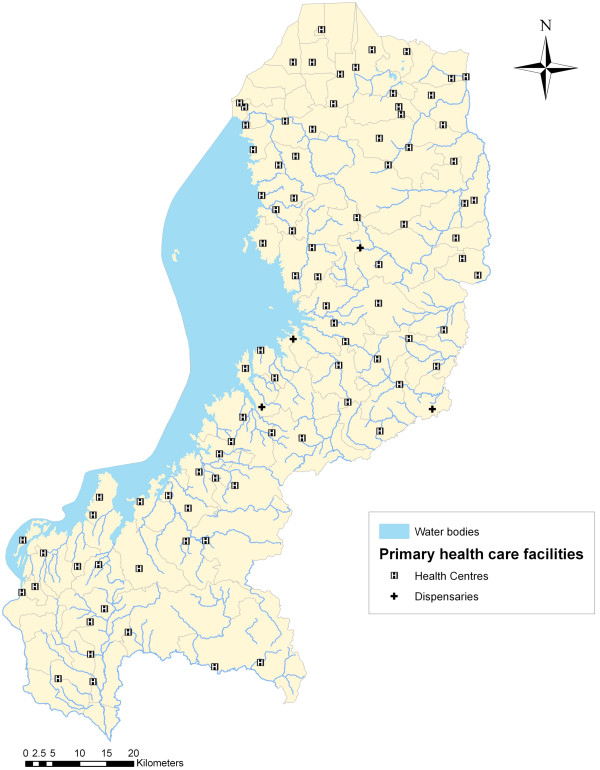
Primary health facilities in the Western Province.

The Rwanda Health Facility Database (RHFDb) was downloaded from the Ministry of Health of Rwanda website ( http://www.moh.gov.rw). There were a total of 113 PHC facilities in the WP, which include health centres, health posts and dispensaries. Facilities without coordinates and/or population coverage capacity (the so-called “population cible de la zone de rayonnement”, i.e. catchment population) data were excluded from the analysis. Nine health facilities (8.0%) did not have coordinates and catchment population (2 health centres, 1 health post, and 6 dispensaries). In addition, seven health centres (6.2%) were missing spatial information and an additional three (2.7%) did not have data on catchment population. The total number of health facilities included in the analysis is 94 (83.2%) and includes 91 health centres and 3 health dispensaries (Figure 1). After superimposing the facilites onto the final landcover gird, three facilities were located on cells considered to be water bodies. These facilities were manually moved to the nearest cell. In addition, Google Earth was used to make sure the facilities were moved to the correct side of the river.

### Population data

Population data at the sector level (third-level administrative sub-division) in the WP was personally obtained from a demographic and socioeconomic survey carried out in 2008 by the National Institute of Statistics [21]. A gridded population distribution map was created by using dasymetric mapping and incorporating areal weighting and empirical sampling techniques. This was used to assess the relationship between categorical landcover data and population distribution [24-26]. Dasymetric mapping is a type of areal interpolation that disaggregates spatial data to a finer unit of analysis using ancillary data to help refine locations of populations or other phenomena being mapped. Areal weighting is a technique where each grid cell is assigned a population value based on its percentage area of the host areal unit [25]. Empirical sampling provides a proportional density fraction used as a weighted value representative of each land class to account for the relative densities in each land category. To obtain the necessary population density values, administrative-level sectors that are entirely occupied by a single inhabited land-cover class are isolated and the population density is calculated by dividing total population by total area. These density ratios are extrapolated to all sectors that have a combination of landcover inhabited classes. Area ratios are then calculated to adjust the population density fraction by the percentage of that block group’s total area that the land category covers [25]. The original population values were redistributed into a surface grid based on thirteen landcover classes and three road categories using ArcGIS (ESRI Inc., Redlands, USA, version 9.3) and dasymmetric mapping [27]. Additionally, following the approach of Sleeter [24], an exclusion class representing zero population was assigned to water bodies and roads. To avoid an automatic correction that will resample the final landcover and the elevation grid using the nearest neighbour interpolation and the bilinear interpolation, respectively, the resolution for the population grid was set at 90 m.

### Ancillary data

The administrative boundaries at the sector level were obtained from the RHBDb. The Digital Elevation Model (DEM), the road and river networks as well as the landcover layer were obtained from the Centre for Geographical Information Systems – National University of Rwanda. The DEM from the Shuttle Radar Topographic Mission has a 3-arc sec (90 meter) resolution. The area covering the WP was extracted by using a mask of the WP. The road network was reclassified into three categories to reflect the Government of Rwanda road classification system [28]: 1) national roads; 2) district roads; and 3) rural/feeder roads. The landcover dataset contained 29 individual classes which were reclassified and aggregated to a more generic 13 classes based on the Global Accessibility Map project of the Global Environment Monitoring Unit of the European Commission Joint Research Centre [29]. The landcover, the road and river network layers were rasterized to match the resolution of the DEM and the population grid and merged to a final landcover grid. The road network was merged at the end so that the road data represent bridges that certainly exist but for which no information is available.

### Analysis

#### Travel time distribution grid (Geographical Accessibility)

The creation of this grid first required the definition of the different travel scenarios utilized by the population to attend the nearest primary health facility. In the context of this paper, we have considered three travel scenarios in which patients are travelling towards the health facilities (Table 1). The use of different travel speeds for different land cover or land use classes is recommended [14]. Land cover classes were assigned travel speeds based on a global map of accessibility [29]. In Scenario 1, all patients are walking to the nearest primary health centre. Walking is the predominant form of transportation in rural Africa as a result of the lack of infrastructure and motorized transport services [30]. Based on recommendations, a mean walking speed on flat surface of 5 km/h was set [14]. Scenario 2 assumes that patients first walk to the nearest road and then use a bicycle to continue their journey. Rwanda is one of a few countries in SSA where bicycles have achieved widespread use, even in the mountainous regions of the country [31]. A mean bicycling speed on flat surface of 10 km/h was used [14]. The third travel scenario considers patients walking to the nearest national or district road and then continue on using a minibus since it is the most common mode of public transport in most African cities [30]. Travelling by minibus only applies to national and district roads in the district of Gisenyi. Speed on these roads was assigned based on the Rwanda national guidelines for roads [28]. The maximum travelling time permitted was set at 60 minutes, in agreement with the MoH norm that the population should have access to a health facility within one hour of walking [32].

**Table 1:** Travel scenarios to the health centre

**Landcover type**	**Travel speeds (km/hr)[**[14]**,**[29]**]**				
	**Scenario 1**	**Scenario 2**		**Scenario 3**	
	**Walking**	**Walking**	**Cycling**	**Walking**	**Public transport**
Mosaic: Cropland/Shrub and/or Grass Cover	1.67	1.67	-	1.67	-
Open/Closed Evergreen/Deciduous Shrub Cover	1.67	1.67	-	1.67	-
Regularly Flooded Shrub and/or Herbaceous	1	1	-	1	-
Sparse Herbaceous or Shrub Cover	2.5	2.5	-	2.5	-
Tree Cover: Broadleaved, Deciduous, Open	1.25	1.25	-	1.25	-
Artificial and Associated Areas	5	5	-	5	-
Mosaic: Cropland/Tree Cover/Other Natural Vegetation	1.67	1.67	-	1.67	-
Tree Cover: Needle-leaved, Evergreen	1.67	1.67	-	1.67	-
Cultivated and Managed Areas	1.67	1.67	-	1.67	-
Mosaic: Tree Cover/Other Natural Vegetation	1.25	1.25	-	1.25	-
Water bodies	NA	NA	NA	NA	NA
Tree Cover: Broadleaved, Evergreen	1	1	-	1	-
Herbaceous Cover, Closed-Open	1.67	1.67	-	1.67	-
National Roads	5	-	10	5	50
District Roads	5	-	10	5	20
Rural/Feeder Roads	5	-	10	5	5

Considering these scenarios, a raster surface of travel time between primary health facilities and population was developed in AccessMod (WHO, Switzerland, Geneva, version 3.0) [33]. The calculation is done through the least-cost path algorithm and takes into account topography of the terrain, landcover, road and river networks, and the corresponding travel speeds through each of the road and landcover classes. Water bodies have been considered as barriers for patients wishing to attend the closest primary health facility. To set this particular landcover category as a barrier and to prevent catchments to cover these areas, the speed of travel was set to 0. The DEM allows the incorporation of slope into the analysis, which is important because the topography of the terrain may accelerate or impede the speed of travelling, especially when walking or cycling. The model, therefore, includes slope-based corrections when walking and cycling based on Tobler’s formula [34] and speed power calculation, respectively [35].

#### Modelling spatial accessibility

This analysis integrates the spatial distribution of the service (supply) and of the population (demand). The catchment area of each facility is calculated by taking into account its population coverage capacity, the population distribution, the terrain topography and the travelling modes through the different land categories. The catchment area is determined by the travel time or the catchment population, whichever is reached first. This means that health facilities that have realized the maximum travel time have not realized their maximum capacity and thus are working below their capacity. Health facilities that have reached their catchment population before reaching one hour of travelling time are operating at their maximum capacity. The model utilizes the least-cost algorithm whereby the location of a health facility is selected as the origin and the maximum travel time of 60 minutes as the limitation for determining the extension of the corresponding catchment area. The model assumes that a patient can only be served by one primary health care facility and that the WP is a closed system (i.e. population cannot be served by health facilities outside the WP and population outside the WP cannot seek care in it) [35].

## Results

### Geographical accessibility to primary health care

**Figure 2:**
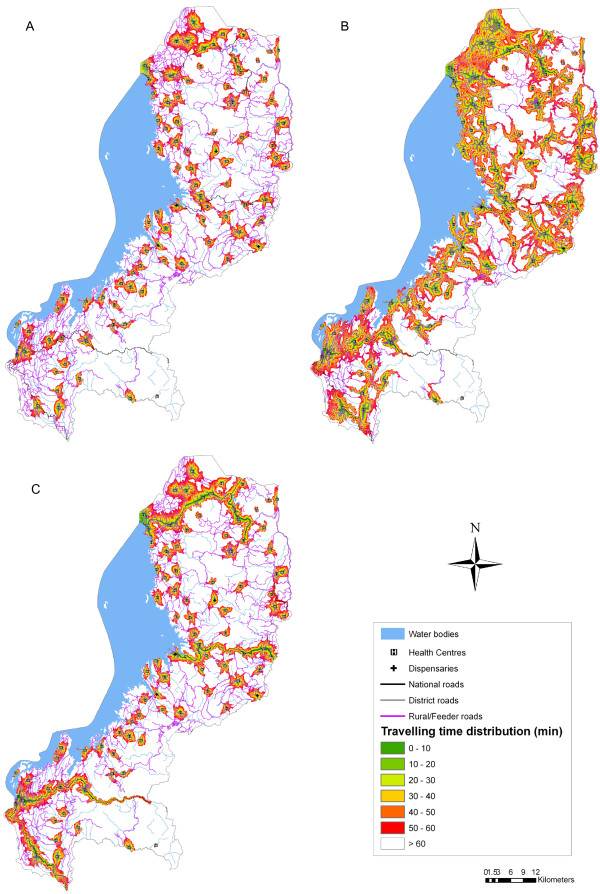
**Geographical accessibility to primary health facilities based on three travel scenarios and a maximum travelling time of 60 minutes.** (**A**) Scenario 1: Walking only; (**B**) Scenario 2: Walking and cycling; (**C**) Scenario 3: Walking and public transport.

This analysis takes into account landscape constraints and was carried out using the three different travel scenarios summarized in Table 1 and a maximum travelling time of 60 minutes. Figure 2 provides a visual representation of the level of accessibility to PHC in the WP. Scenario 1: Walking only (Figure 2a) shows the lowest degree of geographical accessibility. Accessibility is significantly increased when patients first walk to the nearest national or district road and then use public transportation (Figure 2c). The use of a motor vehicle along national and district roads significantly lengthens the travelling distance within the set maximum travelling time. The highest level of geographical accessibility can be observed in Scenario 2: Walking and cycling (Figure 2b). In this case, patients are cycling along the rural/feeder roads, which constitute the majority of the road infrastructure in the WP.

### Spatial coverage of the existing primary health facility network

**Figure 3:**
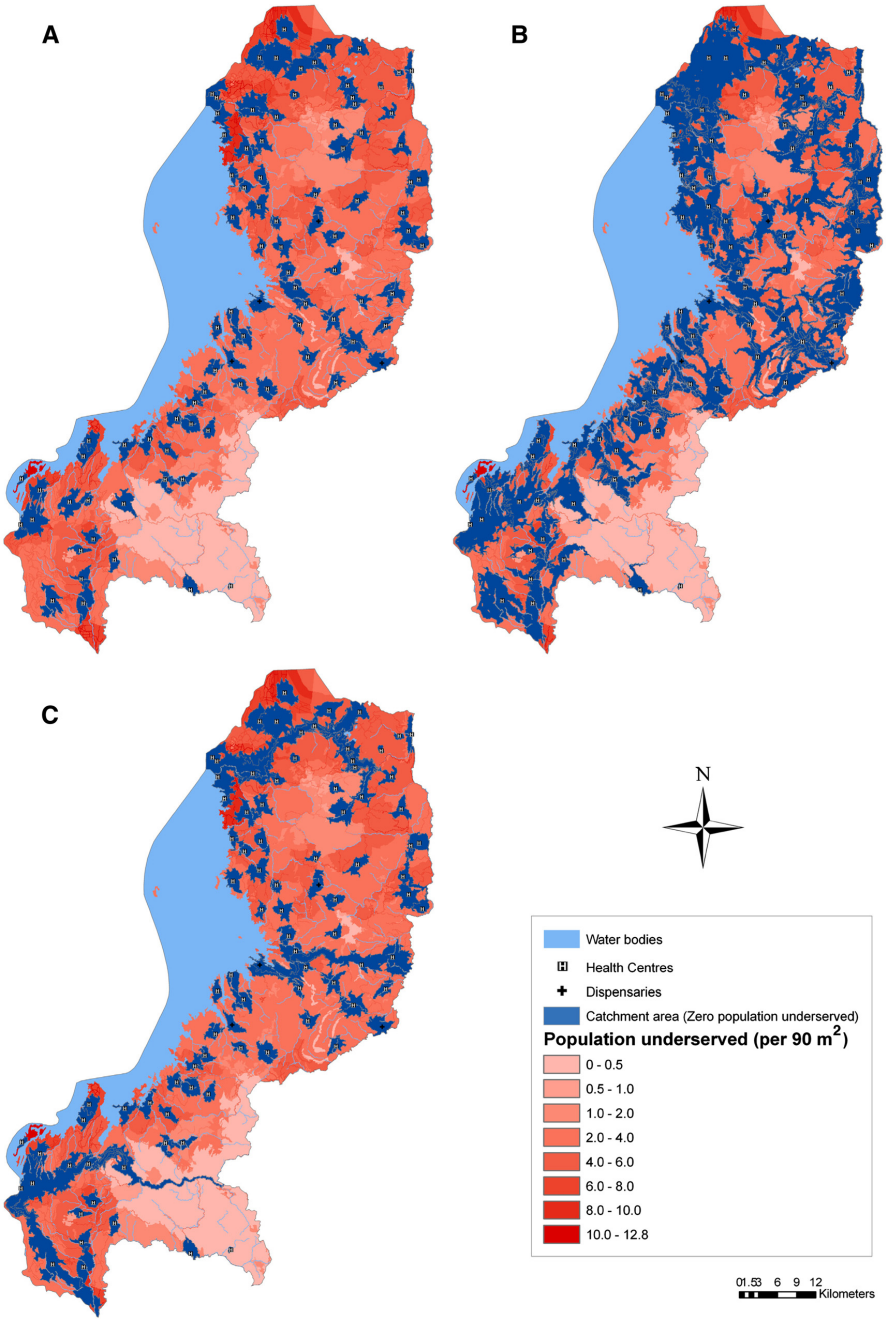
**Extent of catchment areas and distribution of population underserved by the existing primary health facility network based on three travel scenarios and a maximum travelling time of 60 minutes.** (**A**) Scenario 1: Walking only; (**B**) Scenario 2: Walking and cycling; (**C**) Scenario 3: Walking and public transport.

The analysis of the spatial coverage models the extension of the catchment area of each facility. In this analysis, the population coverage capacity of each health facility is considered as the size of the supply and the population distribution grid as the spatial distribution of the demand. Figure 3 shows the extent of the catchment areas based on each travel scenario and a maximum travelling time of 60 minutes. The shape of the catchment areas reflects how the different types of landcover, the road network, and the topography impact travel time. The catchment areas of Scenarios 2 (Figure 3b) and 3 (Figure 3c) extend much further than for Scenario 1 (Figure 3a). The use of a bicycle in Scenario 2 allows patients to travel faster and farther along the extensive network of rural/feeder roads compared to Scenario 1. In Scenario 3, the use of public transportation along national and district roads extends the catchment areas along these routes.

The total population covered also differs depending on the type of travel scenario (Table 2, Figure 3). The existing health facility network covers only 26.6% of inhabitants from the initial 2,091,065 when the only mode of transportation is walking. In Scenario 2, utilizing a bicycle after walking to the closest road, greatly increases the population being served. In this case, the primary health facility network covers 58% of inhabitants. When considering Scenario 3, the total population served is 34.3%.

**Table 2:** Spatial coverage results of the existing primary health facility network based on a maximum travelling time of 60minutes

	**Scenario 1: Walking only**	**Scenario 2: Walking and cycling**	**Scenario 3: Walking and public transport**
Population covered	556,919 (26.6%)	1,212,510 (58.0%)	717,439 (34.3%)
Underserved population	1,534,146 (73.4%)	878,555 (42%)	1,373,626 (65.7%)
Facilities realizing maximum travel time	91 (96.8%)	66 (70.2%)	84 (89.4%)
Facilities realizing maximum capacity	3 (3.2%)	28 (29.8%)	10 (10.6%)

### Percentage of primary health facilities working under capacity

Given a maximum travelling time of 60 minutes, this analysis also demonstrates that in Scenarios 1 and 3, 96.8% and 89.4% of health facilities, respectively, are operating below their maximum capacity utilization (Table 2). This is in contrast with 70.2% of health facilities in Scenario 2. In Scenario 2, 29.8% of health facilities covered their capacity before reaching one hour of travelling time confirming that these facilities are operating at their maximum capacity utilization.

## Discussion

In this study, we investigated geographical accessibility and spatial coverage of the existing primary health facility network in the WP of Rwanda based on a maximum travelling time of 60 minutes. Scenario 2 was the best model covering 58% of the population. Facilities under capacity were also the lowest with only 66 health facilities realizing their maximum travel time. The travelling time to the geographically nearest health facility does not encompass all aspects associated to health care access. The availability or the supply of care provided by the health facility should also be taken into account. Combined measures of demand and supply in the form of accessibility and availability coverage allow us to better understand the causes of poor performance of a health system. Additionally, it enables us to identify factors that prevent the achievement of a desirable level of effective coverage of the population with essential health services [13]. Thus, this study provides a more comprehensive and realistic analysis than methods that take into consideration only one aspect (availability or accessibility coverage). The present research may contribute to a deeper understanding of the performance of the health system and the identification of potential gaps. Thus, the results may represent a useful asset for decision-support in improving health planning and evidence-based policy development.

We can be confident of a number of important findings. The results of the geographical accessibility analysis demonstrates the travelling time to the nearest primary health facility and, therefore, provides a measure of accessibility to PHC. The maximum travelling time for a patient requiring access to a particular primary health facility depends on the severity of the patient’s condition. In line with the Ministry of Health’s (MoH) norm that the population should have access to a health facility within one hour of walking [32] our analysis uses 60 minutes as the maximum travelling time. The analysis takes into account the road network, the different landcover categories, the topography of the terrain, and the travel speeds through each of the roads and landcover classes. The results provide a useful visual summary of the level of accessibility of the WP, where highly and difficult accessible areas can be observed. Scenario 2 has the highest degree of accessibility followed by Scenario 3. The lowest level of accessibility can be observed in Scenario 1 as expected.

The analysis of the spatial coverage calculates the extension of the catchment area of each facility. The spatial extent of the catchment area for each health facility is determined once the maximum population capacity and/or the maximum travel time have been reached. The results provide important evidence that the appropriate travelling time over which to define the catchment population extends beyond the 60-minute time limit. The analysis also demonstrates that the mode of transportation has a significant impact on the served population. While geographical access to health facilities has improved in the last few years [32], when walking is the single mode of transportation considered, only 26.6% of the population in the WP is covered by the catchment area of the existing primary health facility network. Given the results, the MoH’s efforts in ensuring access to a health facility within one hour of walking is far from being realized in the WP. The findings of the present study contrast with a Government of Rwanda study, which suggests that less than 40% of the population still have to walk more than one hour (or more than 5 km) to reach the closest health facility [32]. Combining the use of a bicycle with walking as modes of transportation results in more than a two-fold increase in the number of people covered compared to only walking. The use of a bicycle along the extensive network of rural/feeder roads, which account for over 50% of the road network in the country [28], lengthens the distance patients can travel in one hour, thus, allowing more than half of the population to access PHC. Using public transportation, over one third of the population is covered by the catchment areas of health facilities. The limited road network through which public transportation vehicles can travel restricts access to PHC facilities. The results of the three scenarios underline a lack of PHC services to cover the total population living in the WP.

There exist a number of limitations in this type of analysis that must be considered when interpreting the results. While the present analysis assumes that accessibility is gender neutral, this may not be the case in this particular context. Gender-based inequalities in education, asset ownership, income, and employment as well as women’s lack of decision-making power limit their ability to access and obtain the health care they need [36]. Additionally, women may not have predominant access to bicycles in Rwanda. A second assumption is that patients will always travel to the nearest health facility. However, patients might be inclined to use more distant health care facilities thought to provide higher quality services based, for example, on the availability of drugs and staff among other factors [37]. While recognizing this, attending the nearest health facility can still be considered to be the most common behaviour in the majority of the cases [14]. In terms of population coverage capacity, there exists an underlying assumption that services and resources are always optimal for facilities to realize their maximum capacity. Another assumption is that travel always happens along optimum paths in terms of total travelling time. The estimated travelling time is therefore assumed to be representative of real travelling times. Although based on motorized travel, a study by Haynes et al. [38] found that GIS estimates of car travel times were close to reported times. While some members of the population may use other paths due to habits, social factors, environmental and surface conditions, or other factors, the least-cost approach reflects the overall mode in which people tend to travel [14]. A final limitation in the analysis is the missing data of 19 health facilities, which represent 16.8% of the initial number of facilities. Although it is unknown whether the missing facilities would be working at or below capacity, what is certain is that each health facility would have contributed to an increase in the size of the supply and consequently an increase in the population being served.

## Conclusions

In the present study we have explored important aspects of the PHC system in the WP of Rwanda. Effective health policies require a better understanding of the health system, its functions, and its determinants [13]. The geographical access and the spatial coverage surfaces produced in this analysis provide simple but visually powerful tools that can be used to support health research and decision-making in planning and resource allocation at the district level. The analysis demonstrates significant spatial variations in geographical accessibility and spatial coverage of the primary health system across the three different travel scenarios. Regardless of the mode of transportation, the majority of the population in the WP does not have access to primary health care and more than half of the facilities are working under capacity. Although Rwanda has made substantial efforts in strengthening its primary health system by addressing shortage of health staff, inequity of access, and poor quality of care in health facilities much remains to be done. Increased investment in horizontal care is needed to strengthen the local PHC system and expand coverage. In addition, transport mechanisms to increase geographical accessibility to health care should be enhanced. The results of this study demonstrate that the potential use of a bicycle as a low cost vehicle should not be underestimated. Moreover, our results can also prove valuable in supporting the development of health infrastructure in specific sites to maximize access and reduce inequities. However, given limited transport infrastructure and inadequate resources for running costs of health facilities, it might not be economically feasible to scale up the primary health facility network to a level that will cover the majority of the population. Reducing inequities in access to basic health services might require a more generic and comprehensive approach to organizing the primary health system and could include the expansion of the number of Community Health Workers and the scope of essential health care services delivered at the community level.

Achieving higher quality and cost-effectiveness in the health system as well as improved health outcomes requires a strong primary health care system. Physical accessibiltiy to health care remains a problem in developing countries where large segments of the population live in rural areas. In low income countries primary care programs are effective ways of strenghtening health systems and improve access to health care. Knowledge and understanding of health utilization patters and population distribution are important for the effective delivery of health care. Our findings demonstrate the usefulness of GIS to leverage multiple datasets from different sources in a spatial framework to provide support to evidence-based planning and resource allocation decision-making in developing countries. Additionally, by incorporating information on the demand and supply of care, GIS methods presented in this study can support health planners in identifying potential locations for new primary health facilities where maximum increase in accessibility can be achieved.

## Competing interests

The authors declare that they have no competing interests.

## Authors' contributions

UHM participated in the study design, analyzed data, interpreted the results, and drafted the manuscript. CK made revisions to the manuscript. All authors read and approved the final manuscript.
